# Quantitative assessment of spicule length in *Heligmosomoides* spp. (Nematoda, Heligmosomidae): distinction between *H. bakeri*, *H. polygyrus* and *H. glareoli*

**DOI:** 10.1017/S0031182023000872

**Published:** 2023-09

**Authors:** Mayowa Musah-Eroje, Laura Burton, Nicola Kerr, Jerzy M. Behnke

**Affiliations:** School of Life Sciences, University of Nottingham, University Park, Nottingham, NG7 2RD, UK

**Keywords:** *Apodemus s*pp, *Heligmosomoides bakeri*, *Heligmosomoides glareoli*, *Heligmosomoides polygyrus*, male worms, *Mus* spp, *Myodes glareolus*, spicules

## Abstract

Nematode spicules play a vital role in the reproductive activity of species that possess them. Our primary objective was to compare the lengths of spicules of the laboratory mouse (*Mus musculus*) – maintained isolate *H. bakeri* – with those of *H. polygyrus* from naturally infected wood mice (*Apodemus sylvaticus*). On a more limited scale, we also included *H. glareoli* from bank voles (*Myodes glareolus*), a species reputed to possess longer spicules than either of the 2 former species. In total, we measured 1264 spicules (*H. bakeri*, *n* = 614; *H. polygyrus n* = 582; and *H. glareoli, n* = 68). There was a highly significant difference between the spicule lengths of the Nottingham-maintained *H. bakeri* (mean = 0.518 mm) and *H. polygyrus* (0.598 mm) from 11 different localities across the British Isles. A comparison of the spicules of *H. bakeri* maintained in 4 different laboratories in 3 continents revealed a range in the mean values from 0.518 to 0.540 mm, while those of worms from Australian wild house mice were shorter (0.480 mm). Mean values for *H. polygyrus* from wood mice from the British Isles ranged from 0.564 to 0.635 mm, although isolates of this species from Norway had longer spicules (0.670 mm). In agreement with the literature, the spicules of *H. glareoli* were considerably longer (1.098 mm). Since spicules play a vital role in the reproduction of nematode species that possess them, the difference in spicule lengths between *H. bakeri* and *H. polygyrus* adds to the growing evidence that these 2 are quite distinct species and likely reproductively isolated.

## Graphical abstract

**Figure:**
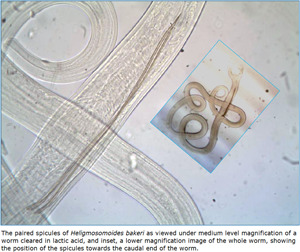

## Introduction

The spicules of male nematodes rank as perhaps their best-known defining features, critically important in the taxonomy of the phylum Nematoda and valuable in distinguishing between genera in the phylum (Crofton, 1966; Kaufmann, 1996), as well as in the identification of individual species (Lichtenfels et al., 1988, 1994). Nematode spicules can be easily studied because when the worms are cleared by appropriate media such as lactic acid, in preparation for microscopical examination, their spicules remain distinct and visible against the translucent background tissues of the worms. Hence, they can be assessed and measured easily and accurately, particularly using currently available digital photography and associated software. Although not all nematode genera possess spicules (e.g. *Aspiculuris* spp. and *Myolaimus* spp.), in those that do, spicules may vary between species in length and shape, and whether they are present as a pair or just as a single spicule. In species that have a pair of spicules, both may be of identical length and shape, and hence symmetrical, or radically different from one another in both respects (Högger and Bird, 1974; Lichtenfels et al., 1988; Smales et al., 2009) and may even be atypical and malformed in some individual worms (Andrews, 1970; Roy and Beveridge, 1997). Relatively few studies have explored the chemical composition of nematode spicules, but unlike the calcareous spicules of other invertebrate phyla (Kingsley, 1984), those of nematodes are thought not to be comprised of calcium as their structural element. Instead, nematode spicules have been shown to consist of a sclerotized protein outer layer containing keratin and collagen, and protoplasmic inner layer with carbohydrates and lipids (Stringfellow, 1971; Clark and Shepherd, 1977; Sood and Kaur, 1983). Spicules are innervated and have sensory functions (Lee, 1973; Wang and Chen, 1985; Emmons, 2008). During copulation, spicules are used to dilate the vulva of female worms against the inner hydrostatic pressure in the pseudocoelomic cavity, ensure subsequent attachment to the females, and then to facilitate transfer of the amoeboid spermatozoa (Barr and Garcia, 2006).

There has been a long debate about whether the dominant trichostrongyloid intestinal nematode *Heligmosomoides polygyrus* (Dujardin, 1845) of the Palearctic wood and yellow-necked mice (*Apodemus sylvaticus* and *A. flavicollis*, respectively*)* and the morphologically similar, laboratory-maintained model species *H. bakeri* (both previously referred to in different papers as *Nematospiroides dubius* and *Heligmosomoides polygyrus*) are the same species or whether they are distinct species in their own right. The history of the isolation of the latter was documented in some detail by Behnke et al. (1991, 2009) and Behnke and Harris (2010), who also argued that based on accepted nomenclature rules, priority for the name *H. polygyrus* is with the naturally occurring parasite in Palearctic *Apodemus* spp. Evidence has been provided for some morphological differences between *H. polygyrus* and *H. bakeri* (Durette-Desset et al., 1972; Behnke et al., 1991), for differences in protein synthesis (Abu-Madi et al., 1994*a*), host specificity (Quinnell et al., 1991), genomic differences (Abu-Madi et al., 1994*b*, 2000) and for base-pair sequence differences in nuclear and mitochondrial genes (Zaleśny et al., 2014; Harris et al., 2015). The laboratory-maintained species is *H. bakeri*, having been classified earlier as a subspecies (*H. polygyrus bakeri*) by Durette-Desset et al., (1972) based on morphology and then raised to species level by Cable et al. (2006) following molecular phylogenetic analysis of key genes, including the ribosomal DNA internal transcribed spacers (ITS) and the mitochondrial cytochrome c oxidase I (*COI*) gene. Despite arguments encouraging acceptance of their separate species status (Behnke et al., 2009; Behnke and Harris, 2010), this has been strongly disputed (Maizels et al., 2011). However, recently, the genomes of both *H. polygyrus* and *H. bakeri* have been sequenced (Stevens et al., under review), and it is now clear that indeed their genomes differ sufficiently for them to be unequivocally regarded as 2 distinct species, with a common ancestor estimated at 1.1–6.4 million years ago (assuming an average of six generations and one generation per year, respectively; Stevens, pers. com.).

Some earlier work has indicated that the spicules of *H. polygyrus* are longer than those of *H. bakeri* (e.g. compare data in Genov and Jančev (1981) for *H. polygyrus* with Durette-Desset et al. (1972) for *H. bakeri*). In view of the importance of spicules in the mating behaviour of nematodes, and the likelihood that differences in spicule length may contribute to the reproductive separation of species, the aim of the current work was to assess quantitatively the range of variation in spicule lengths in each of these 2 species. For this purpose, the spicule lengths of laboratory-passaged *H. bakeri* were compared with those of *H. polygyrus* from wood mice that had been sampled in different parts of the UK and on a more limited scale from other European locations, as well as with isolates of another recognized *Heligmosomoides* species.

## Materials and methods

### Sources of worms for measurement

This work is based on a range of isolates of *Helgmosomoides* spp., some derived from our own fieldwork in different localities across the British Isles and others provided by collaborators in other institutions abroad. *Heligmosomoides bakeri* worms of the strain referred to hereafter as the Australian strain were obtained from Carolyn Behm in 2012. This strain was acquired from Colin Dobson in the 1980s (C. Behm, pers. com.), who originally obtained the strain from the Wellcome Foundation Research Laboratories in London during his PhD studentship at Sheffield University in the UK (Dobson, 1961). The strain hereafter referred to as the Canadian strain was obtained from Marilyn Scott in 2012 and acquired originally from Mike Sukhdeo in the late 1980s (M. Scott, pers. com.). Mike Sukhdeo in turn had obtained infective larvae from John Wetzel of the Ayerst Research Labs New York (Sukhdeo et al., 1984). Although earlier ancestry of this line is unknown, it is most likely to have originated either from the Wellcome Foundation or from Spurlock in the USA (Spurlock, 1943). The worms referred to hereafter as the USA strain were from Debbie Kristan whose cultures at the time (2012) were derived from 2 sources: from Mike Sukhdeo (as above) and from Nedim Ince and Joseph Urban (Beltsville Human Nutrition Research Center, USA). The Beltsville strain was obtained from Lis Eriksen at the Royal Veterinary and Agricultural University in Copenhagen around 1983. Although uncertain, it is likely that this isolate also came originally from the Wellcome Foundation. The Nottingham strain was acquired in 1975 directly from the Wellcome Foundation. It was first maintained for 2 years at Glasgow University and thereafter at Nottingham University until 2014, without any enrichment from other lineages of this species.

The worms and their spicules were measured in 3 separate sessions over 3 successive years (2012, 2013 and 2014), and accordingly, we refer below to Sessions 1–3. The available background details for each batch of worms are given in Table S1. All the material used here had been frozen at −80°C prior to measurement of the spicules, although the duration of freezing was variable, as were also the preservatives used for fixation, the duration of fixation and storage at −80°C (Table S1). The oldest sample was from Australian wild house mice, originally isolated in 1982 and preserved in formalin at room temperature for over 30 years. This isolate was sent to Nottingham in 2012 when it was transferred to 70% ethanol and frozen a day after arrival. Measurements were carried out in the period 2012–2014.

### Procedures used for measurement of spicules

After thawing, individual worms were carefully placed onto a glass slide using fine watch-makers forceps and cleared with 1–2 drops of lactic acid. The worms that had been preserved in formalin took a longer time to clear. The slide was then examined in an Olympus Light Microscope and photomicrographed at × 20, using a high-resolution optic camera and saved in digital format. The worms were measured on photographs using the Image J application, each spicule being measured 4 times and averaged. Each batch of photographed worms was also accompanied by a photograph of a calibration slide (1 mm), and the measurement of this was used to convert the number of counted pixels on photographs of worms to the corresponding length in millimetres. Since there is no consistent difference between each of the 2 spicules of individual worms of these species, and variation is only minimal, in the range 0.36–6.86% (Musah-Eroje et al., 2021*a*), we treated each spicule as an independent datum. Spicule lengths are given as mean ± standard error of the mean (s.e.m.). Measurements are in millimetres (mm) and some instances in micrometres (*μ*m), as stated.

### Statistical analysis

Where possible all datasets were tested for goodness of fit to the Gaussian distribution using bespoke software in Excel based on Elliott (1977). We also calculated the index of dispersion (*I* = variance/mean ratio) and the index of discrepancy (*D*; Poulin, 1993) as additional guides for the pattern of distribution of the data. Where relevant we fitted GLM models in IBM SPSS version 28 (1 New Orchard Road, Armonk, New York 10504-1722, USA) and tested residuals for goodness of fit to the Gaussian distribution. Since some datasets were limited numerically, and a Gaussian distribution could not be tested for, nor assumed, analysis in these cases was based on non-parametric models (Mann–Whitney *U* test and Kruskal–Wallis test for which the test statistic is *H*).

## Results

### Spicule length of *Heligmosomoides bakeri* in mice maintained in Nottingham

**Figure 1.**
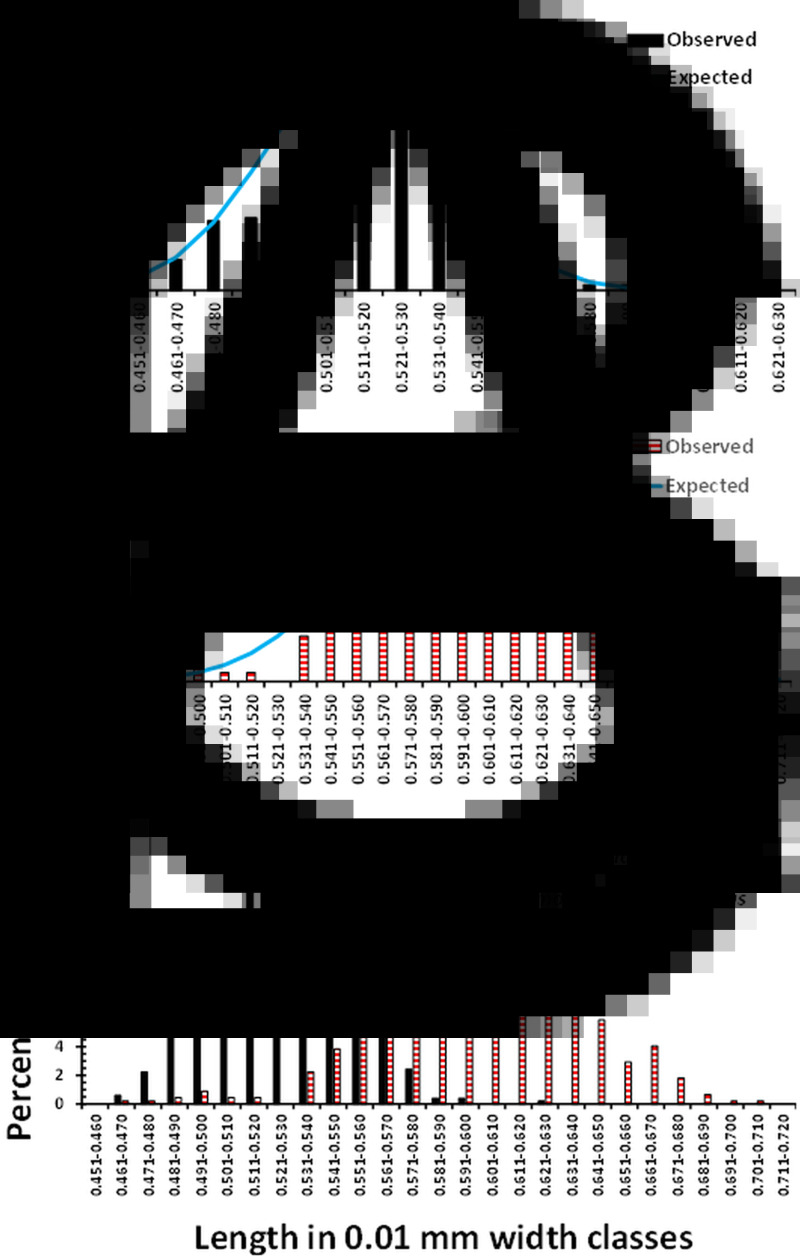
Frequency distribution of the lengths of spicules of *H. bakeri* (Nottingham strain) and *H. polygyrus* (isolates from the British Isles). (a) Observed values of spicule lengths of *H. bakeri* worms from all 3 sessions (*n* = 494, *I* = 0.001, and *D* = 0.183) and those expected of the Gaussian distribution. (b) Observed values of spicule lengths of *H. polygyrus* from 11 localities in the British Isles (combined data, *n* = 444, *I* = 0.003, and *D* = 0.154) and those expected of the Gaussian distribution. (c) Comparison of the distribution of the lengths of spicules of *H. bakeri* and *H. polygyrus* expressed as the percentage of the total of each species, to compensate for the difference in sample sizes.

The mean lengths of spicules and associated summary statistics, recorded from the *H. bakeri* strain, maintained in Nottingham over the 3 sessions are summarized in Table 1. The overall mean spicule length was 0.518 ± 0.0011 (*n* = 494). Despite the very similar mean values obtained in the 3 sessions, differing only by 9 *μ*m, there was a significant difference between these 3 means (*F*_2, 491_ = 5.416, *P* = 0.005), although it only accounted for 1.8% of the variance. The relatively low value obtained in Session 1, differing from that obtained in Session 2 (Tukey's HSD MRT, *P* = 0.003), arose through some of the lower values derived from the 11-day-old worms. The distribution of spicule lengths, in 100 *μ*m length classes, differed significantly from that expected of a Gaussian distribution (Fig. 1a; *χ*^2^_11_ = 33.6, *P* < 0.001), although the values of both *I* and *D* indicated this distribution model as the most appropriate (Fig. 1a).

**Table 1.** Summary statistics for length of spicules of *Heligmosomoides bakeri*

Isolate (age, days)	*n*	Mean length (mm)±	s.e.m.	Range	
				Minimum	Maximum
Session 1					
Nottingham (11)	50	0.506	0.0029	0.463	0.545
Nottingham (13)	50	0.513	0.0028	0.472	0.571
Nottingham (21)^a^	50	0.518	0.0031	0.467	0.561
Nottingham (11, 13, 21)	150	0.512	0.0017	0.463	0.571
Australia^b^	32	0.520	0.0041	0.458	0.559
USA^b^	20	0.528	0.0037	0.502	0.558
Canada (12)	10	0.540	0.0075	0.500	0.567
Session 2					
Nottingham (21)^c^	218	0.521	0.0015	0.451	0.577
USA^b^	20	0.521	0.0035	0.498	0.549
Session 3					
Nottingham (21)^c^	126	0.518	0.0025	0.451	0.611
Combined Nottingham	494	0.518	0.0011	0.451	0.611
Combined USA	40	0.525	0.0026	0.498	0.558
Combined USA + Canada + Australia	82	0.525	0.0023	0.458	0.567
Combined all data	576	0.519	0.0010	0.451	0.611

a These 21-day-old worms were from the same dataset as that reported for this age class by Musah-Eroje et al. (2021*a*).

b Age was not recorded.

c These 21-day-old worms were from different batches measured on different occasions.

### Spicule length of Heligmosomoides polygyrus from different locations in the British Isles

The mean lengths and associated summary statistics for spicules of worms measured from wood mice trapped in eleven localities in the British Isles are shown in Table 2. The overall mean length of spicules from worms from all 11 sites combined was 0.598 ± 0.0019 mm (*n* = 444), with a range from 0.465 to 0.710 mm, and a distribution that differed significantly from that expected of the Gaussian distribution (Fig. 1b; *χ*^2^_18_ = 40.7, *P* = 0.002). However, as we found with *H. bakeri* (above), the values of both *I* and *D* (Fig. 1) indicated that a normally distributed pattern was appropriate.

**Table 2.** Summary statistics for lengths of spicules of *Heligmosomoides polygyrus* from *Apodemus sylvaticus* sampled in various counties in the British Isles

Species	Locality	*n*	Mean length (mm)±	s.e.m.	Range	
					Minimum	Maximum
Session 1						
*H. polygyrus*	Cornwall	16	0.612	0.0068	0.567	0.655
*H. polygyrus*	Dorset	50	0.564	0.0054	0.467	0.662
*H. polygyrus*	Midlothian	22	0.634	0.0085	0.554	0.680
*H. polygyrus*	Norfolk	42	0.615	0.0048	0.552	0.668
*H. polygyrus*	Nottinghamshire	34	0.567	0.0082	0.465	0.668
*H. polygyrus*	Staffordshire	54	0.585	0.0034	0.535	0.633
Session 2						
*H. polygyrus*	Aberdeenshire	20	0.626	0.0107	0.551	0.710
*H. polygyrus*	Anglesey	20	0.613	0.0046	0.585	0.645
*H. polygyrus*	Dorset	20	0.600	0.0082	0.550	0.682
*H. polygyrus*	Durham	20	0.591	0.0062	0.551	0.638
*H. polygyrus*	Lincolnshire	16	0.593	0.0093	0.550	0.651
*H. polygyrus*	Nottinghamshire	50	0.604	0.0049	0.551	0.668
*H. polygyrus*	Midlothian	20	0.627	0.0061	0.586	0.677
*H. polygyrus*	Mid. Ireland^a^	12	0.635	0.0080	0.583	0.679
Session 3						
*H. polygyrus*	Anglesey	30	0.583	0.0046	0.543	0.644
*H. polygyrus*	Nottinghamshire	18	0.617	0.0049	0.581	0.644
Combined data		444	0.598	0.0019	0.465	0.710

a Theses isolates were from wood mice caught in several locations in Mid Ireland as follows: Rhode, Offally; Vicarstown, Laois; Brackagh, Offally; Slieve Bloom Mts, Laois.

Because of limited sample sizes from some locations, only the spicule lengths of worms from Norfolk (*χ*^2^_3_ = 2.53, *P* = 0.47), Staffordshire (*χ*^2^_7_ = 6.64, *P* = 0.47), Dorset (*χ*^2^_7_ = 10.59, *P* = 0.158) and Nottinghamshire (*χ*^2^_11_ = 21.10, *P* = 0.032) could be tested for goodness of fit to the Gaussian distribution. While the first 3 showed good concordance to the Gaussian distribution, the spicule lengths of worms from mice trapped in Nottinghamshire deviated significantly. However, in all 4 cases, the values of *I* (0.002, 0.001, 0.003 and 0.003, respectively) and *D* (0.244, 0.232, 0.201 and 0.166, respectively) indicated that the Gaussian distribution was acceptable. The lowest mean value was for the spicules of mice from Dorset recorded in Session 1, although the mean value obtained in Session 2 was higher (Table 2). The longest mean spicule length was for the spicules of the mice from Mid. Ireland, representing a 12.6% (71 *μ*m) increase in mean length, relative to the value for spicules from Dorset mice recorded in Session 1. The difference in spicule lengths between the eleven isolates was significant (*F*_10, 443_ = 11.233, *P* < 0.001), accounting for 18.8% of the variance in this model.

### Comparison of the length of spicules of *H. bakeri* (Nottingham lab strain) and *H. polygyrus* from sites in the British Isles

Figure 1c compares the distribution of the spicule lengths of *H. bakeri* and *H. polygyrus,* expressed in percentage terms because of the difference in sample sizes. The distribution of *H. bakeri* spicule lengths spanned a more limited range than that of *H. polygyrus* (variance = 569 × 10^−6^ and 1569 × 10^−6^, respectively), as might be expected from a laboratory-maintained isolate of known age, compared to the wild, naturally breeding *H. polygyrus* isolates of unknown age. Although there was some overlap of lengths, the majority of spicules from *H. polygrus* were longer than those recorded from *H. bakeri.* We recorded 1 outlier among the *H. bakeri* from Nottingham, at 0.611 mm, and 2 with lengths of 0.589 and 0.580 mm in Session 3, but otherwise, the longest spicule was 0.577 mm. Consistent with the fitted Gaussian bell-shaped curve in Fig. 1a, taking this value as the longest reliable *H. bakeri* spicule, 69.4% of the spicules from *H. polygyrus* exceeded this value. Moreover, 97.7% of spicules were longer than the mean value of *H. bakeri* spicules (0.519 mm), and standard deviations (s.d.) did not overlap (for *H. bakeri* mean + 1 s.d. = 0.541 mm and for *H. polygurus*, mean – 1 s.d. = 0.559 mm). There was a highly significant difference between the lengths of *H. bakeri* spicules (Nottingham laboratory strain) and those from British Isles isolates of *H. polygyrus* (*F*_1, 936_ = 1467.6, *P* < 2.2 × 10^−16^), accounting for 61.0% of the variance in this model.

### Spicule length of *Heligmosomoides bakeri* in mice maintained in other laboratories

Table 1 also summarizes the results of measurements of the spicules of *H. bakeri* maintained in laboratory mice in 3 other laboratories, 1 in each of Australia, Canada, and the USA. The spicules from worms from the laboratory in California were measured separately in 2 sessions, but the means only differed by 7 *μ*m (Table 1), and the difference was not significant (*F*_1, 38_ = 2.18, *P* = 0.15). We compared the data for spicules from all 4 laboratories (with sessions combined), and there was a significant difference between the 4 laboratories (*F*_3, 572_ = 4.094, *P* = 0.007), although it only accounted for 1.6% of the variance. The mean values for worms from the other 3 laboratories were all higher than that for the worms from Nottingham, but significance was primarily attributable to the difference in mean values between the shortest (Nottingham) and the longest (Canada) which was only 22 *μ*m (*post hoc* comparison between labs, Tukey's HSD, *P* = 0.014). However, when combined, the mean length of the spicules from the other laboratories was numerically 1.36% longer, their combined mean differing by 7 *μ*m from the combined Nottingham mean (Table 1). This difference was significant (*F*_1, 574_ = 6.371, *P* = 0.012) although it only accounted for 0.9% of the variance.

### Spicule length of *Heligmosomoides* spp. from different locations abroad

Table 3 shows summary statistics for spicule lengths of a range of other isolates that we were able to incorporate into this study. These include *H. bakeri* recovered from wild house mice from Australia in 1982 and *H. polygyrus* isolates from wood mice from Portugal and Norway, and from yellow-necked mice from Italy.

**Table 3.** Summary statistics for lengths of spicules of *Heligmosomoides* spp. from *Mus musculus* and *Apodemus* spp. from various locations abroad and from *Myodes glareolus* from Anglesey in Wales

Species	Locality	Host	*n*	Mean length (mm)±	s.e.m.	Range	
						Minimum	Maximum
Survey 1							
*H. bakeri*	Australia	*M. musculus* (wild)	12	0.463	0.0152	0.385	0.527
*H. polygyrus*	Italy	*A. flavicollis*	38	0.589	0.0059	0.509	0.645
*H. polygyrus*	Portugal	*A. sylvaticus*	40	0.558	0.0063	0.456	0.617
*H. glareoli*	Wales^a^	*M. glareolus*	12	1.078	0.0122	1.019	1.145
Survey 2							
*H. bakeri*	Australia	*M. musculus* (wild)	26	0.488	0.0034	0.450	0.519
*H. polygyrus*	Norway	*A. sylvaticus*	20	0.670	0.0095	0.604	0.737
*H. polygyrus*	Portugal	*A. sylvaticus*	40	0.581	0.0039	0.551	0.650
*H. glareoli*	Wales^a^	*M. glareolus*	20	1.066	0.0136	0.957	1.153
Survey 3							
*H. glareoli*	Wales^a^	*M. glareolus*	36	1.122	0.0087	1.017	1.260
Combined *H. bakeri*			38	0.480	0.0055	0.385	0.527
Combined *H. polygyrus*			138	0.589	0.0043	0.456	0.737
Combined *H. glareoli*			68	1.098	0.0071	0.957	1.260

a The spicules from worms of these isolates were from wood mice from the island of Anglesey in Wales.

The mean value of the spicules from Australian wild house mice was the shortest in our study. The 2 means, from Sessions 1 and 2, differed marginally (*F*_1, 36_ = 4.642, *P* = 0.038) and in both sessions were low even relative to our combined value for *H. bakeri* (0.519 mm). The combined mean value for these spicules (0.480 ± 0.0055) was evidently smaller than any of the means of the other datasets in Table 1 (e.g. 7.46% shorter than the mean for the Nottingham datasets combined).

Spicule lengths of worms from Italy, Portugal and Norway differed significantly (*H*_2_ = 48.8, *P* < 0.001). The longest *H. polygyrus* spicules were recorded for worms from Norwegian wood mice and the shortest for worms from Portuguese wood mice (especially those measured in Session 1). The difference between the spicule lengths of worms from these 2 countries was highly significant (*U*_80, 20_ = 1561.0, *P* < 0.001, and differing in mean length by 100.8 *μ*m). The spicules of worms from Italy were also significantly longer than those from Portugal (*post hoc* test with Bonferroni correction for multiple tests, *P* = 0.044, but differing in mean length by only 10.2 *μ*m). However, all the mean values for the spicules of *H. polygyrus* among these continental isolates, and their combined value, (Table 3) were longer than that for our combined value for the laboratory-maintained strain of *H. bakeri* (overall mean differing by 70.9 *μ*m, *F*_1,712_ = 603.2, *P* < 2.2 × 10^−16^, *R*^2^ = 0.458). Moreover, they were mostly similar to those of *H. polygyrus* from wood mice from localities in the British Isles, the overall means [combined value for worms from the British Isles (Table 2) vs that for combined value for worms from Norway, Portugal and Italy (Table 3)] differing by only 8.9 *μ*m, albeit significantly (*F*_1,580_ = 4.7, *P* = 0.031 and *R*^2^ = 0.006).

### Spicule length of *Heligmosomoides glareoli* from bank voles

We had a limited opportunity to measure the spicules of another species of *Heligmosomoides*, constrained by a small sample size. The summary stats are given in Table 3 and show that as expected the spicules of *H. glareoli* were almost twice as long as those of *H. bakeri*, as documented in the literature (combined mean = 1.098 ± 0.0071, *n* = 68). The 3 means obtained in the different sessions (Table 3) all exceeded 1 mm, but differed significantly (*F*_2, 65_ = 8.028, *P* < 0.001), the model explaining 17.3% of the variance. The difference between sessions was attributable to the slightly longer measurements recorded in Session 3.

## Discussion

The principal aim of the current work was to determine whether the lengths of the spicules of *H. bakeri* differ from those of *H. polygyrus*. Given the crucial function of nematode spicules in mating activity, a significant difference between the spicule lengths of these 2 species could lead to reproductive separation and isolation, and hence explain their separate species status. Indeed, our analysis revealed a clear significant difference between the spicule lengths of the Nottingham-maintained strain of *H. bakeri* and the pooled value of spicule lengths of worms from wood mice sampled in 11 different localities in the British Isles.

The data provided in this report for spicule lengths of the *Heligmosomoides* spp. that we measured and variation in these measurements, based on quantitative measurements (overall a total of 1264 spicules were measured) and associated analysis, contrast with information available in the public domain. For the most part, data in the literature either provide a mean spicule length or a range for a given species (Lichtenfels et al., 1994) and without any information on how many spicules were measured. Thus, the extent of variation within the populations that were sampled has not always been taken into account.

The mean value we obtained for *H. bakeri* (0.519 mm) fits well with the range given by Durette-Desset et al. (1972; 0.420–0.520 mm), although in our case the range was wider (0.451–0.611 mm). However, as pointed out earlier, the highest value we recorded (0.611 mm) was very much an outlier, and 0.577 mm would seem to be a more reliable upper limit. In comparison, the mean spicule length that we recorded for *H. polygyrus* isolates from wood mice from the British Isles was 0.598 mm. This value is very close to the 0.58 mm given by Dujardin (1845) for the spicules of *Strongylus polygyrus* ( = *H. polygyrus*) in the original description of this species and fits the range (0.54–0.6 mm) given by Baylis (1928) for worms (which he referred to as *Nematospiroides dubius*) from wood mice from a locality near Oxford. Our results are also compatible with the range (0.516–0.620 mm) given by Genov and Jančev (1981) and Genov (1984), and the mean provided by Schulz (1926; 0.608 mm). As with *H. bakeri*, in our study, the recorded range of spicule lengths for *H. polygyrus* was wider (0.565–0.710 mm) than reported in these publications.

The smaller variance of spicule lengths of *H. bakeri* compared with *H. polygyrus* is to be expected, given that this species has been passaged in laboratory mice for over 60 years. Ehrenford (1954) isolated the species from wild *Peromyscus maniculatus* in California in 1950 and established a laboratory line (strain 50) which Spurlock then distributed to various laboratories including the Wellcome Foundation in London (Behnke et al., 1991). The strain maintained in Nottingham was obtained from the Wellcome Foundation in 1975 and has been passaged routinely since then, initially at Glasgow University, and from 1976 at Nottingham University in CFLP strain mice. Given the long period of laboratory maintenance, a degree of inbreeding and hence loss of genetic diversity might be expected. However, there is evidence for some degree of genetic variation within laboratory-maintained strains of *H. bakeri* because lines varying in their sensitivity to the host response, and capacity to cause chronic infections, can be created by selective breeding (Dobson and Tang, 1991; Tang et al., 1995). Chehresa et al. (1997) found that separate passage of lines without mixing, resulted within 10 generations in distinct lines differing in aspects of their life histories, while Njoroge et al. (1997) reported selection of lines for enhanced resistance to the anthelmintic drug ivermectin. Moreover, the recent whole genome sequencing of *H. bakeri* has confirmed that there is indeed an unexpectedly high degree of genetic diversity in the Edinburgh strain (acquired from Nottingham in the 1990s; Hewitson et al., 2011) used for this work (Stevens et al., under review). On this basis, it is perhaps not surprising that our analysis of spicule lengths of worms maintained in 4 laboratories, located on 3 continents, revealed significant variation between these strains, especially between the Nottingham and Canadian strains.

Our analysis showed that there was some variation in the spicule lengths of *H. polygyrus* from different regions of the UK and also between worms from hosts from 3 European countries. Variation in nematode morphology has been attributed to various factors in the past, including the age of nematodes (Bryant 1973). In preparation for the current analysis, we followed daily changes in spicule length during the development of *H. bakeri* after experimental infection and found that spicule length does vary somewhat during the development and growth of the worms (Musah-Eroje et al., 2021*a*). Spicules are first detectable by microscopy on day 6 post infection (PI) when the worms are at a late L4 stage of development and moulting to the adult stage. Their length then increases to peak on day 7 PI before contracting by approximately 0.1 mm (from day 7 peak means of 0.59 and 0.61 mm in 2 experiments) until they stabilize by 14 days PI (0.51 and 0.53 mm; Musah-Eroje et al., 2021*a*). Whilst it was not possible for ethical reasons to repeat such experiments in wood mice infected with *H. polygyrus*, the development of worms in this host/parasite combination is unlikely to differ radically. It is conceivable therefore that some of the shorter *H. polygyrus* spicules measured in the current work, whose length overlapped with the longer *H. bakeri* spicules, may have been from recently moulted adult worms. Nevertheless, given the longevity of both *H. bakeri* and *H. polygyrus* (Robinson et al., 1989; Gregory et al., 1990), and the known accumulation of worms with increasing host age (Elton et al., 1931; Behnke et al., 1999), the probability of selecting such worms in large scale sampling, as in the current work, is likely to be very low, although not improbable.

The *H. polygyrus* worms utilized in the current study were from wood mice trapped during fieldwork in various locations over a period of many years and had been preserved mostly in 80% ethanol and frozen at −80°C. It is possible that some of the variation detected in spicule length was attributable to differences arising from the use of different preservatives and associated variation in storage time at −80°C. In an earlier paper, we investigated how the use of standard preservatives might affect spicule length (Musah-Eroje et al., 2021*b*) and concluded that the spicules of *H. bakeri* are robust and change very little in preservatives over time. The maximum change was 5.03% shrinkage in Hanks' balanced saline combined with freezing at −80°C, but shrinkage/expansion in other combinations of preservatives with storage at room temperature or freezing was less. The maximum period of storage in these experiments was 4 months, and it is conceivable that longer periods of storage may have generated greater shrinkage or expansion of spicule length. The *H. bakeri* worms from Australian wild *M. domesticus*, measured in the current work, had shorter spicules than the laboratory-maintained *H. bakeri*, but these worms had been stored in formalin for over 40 years, which may have accounted for the difference. Nevertheless, in our experiments storage in formalin, whether at room temperature or at −80° C, resulted in less than 3% change in length, albeit over periods of only 1 and 4 months.

Among samples from the British Isles, the longest spicules of *H. polygyrus* were from wood mice from Scotland and Mid. Ireland, but the longest of all were from abroad in Norway, perhaps suggesting a latitudinal trend for longer spicules with more northerly latitudes. Some degree of between-locality variation is to be expected, as wood mouse populations across the UK and Europe are not entirely panmictic, with many local barriers to gene flow across the region, including rivers, cities, farmland and even the North Sea in the case of Norway. In W. Europe, *H. polygyrus* populations are known to consist of at least 5 distinct genetic variants represented by 5 clades based on differences in the mitochondrial *cyt b* gene (Nieberding et al., 2005, 2008). Although worms from the United Kingdom were not included in these studies, those from Ireland and Denmark formed Clade 5, which differed significantly from Clade 1, comprising worms from Italy, and Clade 2, those from the Iberian Peninsula. It is therefore possible that to some extent the differences that we found in spicule length between isolates of *H. polygyrus* from localities in the British Isles, as well as between the isolates from the 3 European countries, were attributable to morphological differences between these clades, although the latter were not explored in the work of Nieberding et al. (2005).

Both the mean value that we obtained for the spicule length of *H. glareoli* (1.098 mm) and the range (0.957–1.260 mm) fit well the ranges given by Baylis (1928; 0.8–1.0 mm), Genov and Jančev (1981), Genov (1984; 0.984–1.179 mm), and Biserkov et al. (1998; 0.87–1.0 mm) and confirm that the spicule length of this species is almost twice those of *H. bakeri* and *H. polygyrus*.

In conclusion, in this paper, we have provided summary data (mean lengths and extent of variation in lengths) based on quantitative measurements of the spicules of male *H. polygyrus* (*n* = 582), and *H. bakeri* (*n* = 614) as well as *H. glareoli* (*n* = 68). While *H. polygyrus* and *H. bakeri* are morphologically very similar and cannot be readily distinguished by conventional microscopy, the spicules of male worms of these species differ significantly in length with only minimal overlap in measurements when large sample sizes are assessed. Although we cannot exclude entirely an environmental effect on spicule length (*H. bakeri* develop in *Mus* spp. while *H. polygyrus* in *Apodemus* spp., but see Quinnell et al., 1991), we nevertheless consider that our results are compatible with other recorded differences between these isolates (see above) and especially the recent genomic data indicative of separate species status (Stevens et al., under review). Since spicules play a vital role in reproduction, this difference in spicule length between the species therefore helps to consolidate evidence that indeed *H. bakeri* and *H. polygyrus* are 2 distinct species.

## Supporting information

Musah-Eroje et al. supplementary material Musah-Eroje et al. supplementary material

## Data Availability

All the data analysed in the current paper can be made available on request to JMB.

## Supplementary material

The supplementary material for this article can be found at https://doi.org/10.1017/S0031182023000872
