# Supplementary material for: Quantitative assessment of spicule length in Heligmosomoides spp. (Nematoda, Heligmosomidae): distinction between H. bakeri, H. polygyrus and H. glareoli
Source: Parasitology. 2023 Sep 14;150(11):1022–30. doi: 10.1017/S0031182023000872 (PMC10941215; doi:10.1017/S0031182023000872)
Supplement: Musah-Eroje et al. supplementary material [file S0031182023000872sup001.docx]

**Supporting Information**

**Table S1.** The sources of parasites and their treatment prior to measurement.

| **Isolate** | **Host** | **Source** | **Year*** | **Treatment** |
| --- | --- | --- | --- | --- |
| *H. bakeri* | Laboratory mice | Nottingham University | 2011 | CFLP mice. Worms fixed in 80% ethanol and frozen |
| *H. bakeri* | Laboratory mice | Canada, McGill University | 2012 | 12 day old worms raised in CD-1mice after infection with 100-200 L3, fixed in formalin, then stored in 80% ethanol at -80°C |
| *H. bakeri* | Laboratory mice | Australia, Canberra | 2012 | CBA mice, fixed in 80% ethanol and frozen at -80°C |
| *H. bakeri* | Laboratory mice | USA, California | 2012 | Fixed in 10% formalin and the frozen in 80% ethanol |
| *H. bakeri* | *Mus domesticus* | Australia, New South Wales | 1982 | Fixed in 10% formalin since 1982, 50% ethanol in 2012 for transport, and then frozen in -80°C in 80% ethanol |
| *H. polygyrus* | *Apodemus sylvticuas* | Aberdeenshire | 1997 & 2013 | Fixed in 80% ethanol and frozen in 80% ethanol |
| *H. polygyrus* | *Apodemu sylvaticus* | Cornwall | 2011 | Fixed in 80% ethanol and frozen in 80% ethanol |
| *H. polygyrus* | *Apodemus sylvticuas* | Dorset | 2011 | Fixed in 80% ethanol and frozen in 80% ethanol |
| *H. polygyrus* | *Apodemus sylvticuas* | Durham | 2006 | Fixed in 80% ethanol and frozen in 80% ethanol |
| *H. polygyrus* | *Apodemus sylvaticus* | Lincolnshire | 2000 | Fixed in 80% ethanol and frozen in 80% ethanol |
| *H. polygyrus* | *Apodemus sylvaticus* | Midlothian | 2009 | Fixed in 80% ethanol and frozen in 80% ethanol |
| *H. polygyrus* | *Apodemus sylvaticus* | Norfolk | 2012 | Fixed in 70% ethanol and frozen in 80% ethanol |
| *H. polygyrus* | *Apodemus sylvaticus* | Mid. Ireland | 2011 | Fixed in 10% formalin and frozen in 80% ethanol |
| *H. polygyrus* | *Apodemus sylvaticus* | Nottinghamshire | 2011 & 2013 | Fixed in 80% ethanol and frozen in 80% ethanol |
| *H. polygyrus* | *Apodemus sylvaticus* | Portugal, Pancas | 1991 & 2012 | Fixed in 80% ethanol and frozen in 80% ethanol |
| *H. polygyrus* | *Apodemus sylvticuas* | Staffordshire | 2000 | Fixed in 80% ethanol and frozen in 80% ethanol |
| *H. polygyrus* | *Apodemus flavicollis* | Italy | 2012 | Fixed in 90% ethanol and frozen in 80% ethanol |
| *H. glareoli* | *Myodes glareolus* | Wales, Anglesey | 2009,2010 & 2011 | Fixed in 80% ethanol and frozen in 80% ethanol |

* Year in which the wood mice were caught and worms extracted, and for laboratory mice the year in which they were infected and autopsied for worm recovery.
